# Many Keys Unlock the Doors for Virus Entry

**DOI:** 10.1128/mbio.00445-22

**Published:** 2022-05-17

**Authors:** Tom Gallagher

**Affiliations:** a Department of Microbiology and Immunology, Loyola University Chicago, Maywood, Illinois, USA

**Keywords:** virus entry, endosome, membrane fusion, coronavirus, influenza virus

## Abstract

To successfully infect, viruses must respond to cues that promote their genome delivery into host cells. These keys to virus entry frequently reside inside endocytic vesicles. In a recent mBio article, Poston et al. (D. Poston, Y. Weisblum, A. Hobbs, and P. D. Bieniasz, mBio 13:e0300221, 2022, https://doi.org/10.1128/mbio.03002-21) identified and characterized protein complexes generating endocytic environments favorable for virus entry. These included retromer-associated vacuolar protein sorting 29 (VPS29) proteins. Without VPS29, endosomes lacked cathepsin activities, making them incapable of supporting those viruses in which endosomal proteolysis triggers entry. These protease-dependent viruses encompass several zoonotic filoviruses and coronaviruses, including recent SARS-CoV-2 variants of concern. The valuable findings of Poston et al. reveal retromer complexes as master keys for select endosomal virus entry processes and raise the possibility that threatening coronaviruses might be resisted through targeted inactivation of components controlling endosome structure and function.

## COMMENTARY

As obligate intracellular parasites, viruses depend on a wide assortment of host cell components for their propagation. Identification of these host cell dependency factors brings new insights into virus-host interactions and can also launch new antiviral research; targeting dependency factors with pharmacologic agents can provide therapeutic antiviral activities. For these reasons, virologists are continuously aiming to identify new host cell dependency factors.

Genetic screens have rapidly become a standard approach to identifying host dependency factors. In CRISPR loss-of-function screens, cell populations with diverse edited genes are infected with lytic viruses, leaving small numbers of surviving cells with identifiable knockouts in genes encoding dependency factors. In the past 2 years, these CRISPR screens were employed to recognize host factors facilitating SARS-CoV-2 and related human coronavirus (CoV) infections. Among the commonly identified dependency factors were those controlling endosome functions (1). These findings aligned with those obtained nearly a decade ago in which endocytosis-associated proteins and endosomal maturation processes were discovered as central requirements for infection by the prototype mouse hepatitis CoV (2). The CoV susceptibility factors operated at the level of cell entry (3). More recently, using similar loss-of-function CRISPR screens, Poston et al. (4) identified similar endosome-controlling proteins as human (H)-CoV-OC43 dependency factors. These included components of several complexes, including CCC/Commander, WDR81/91, retromer, and retriever, which together control endosome morphology and maturation, as well as cargo trafficking between endosomes and several other subcellular organelles.

That these dependency factors were key hits in several CRISPR screens may have been predicted. During cell entry, many disparate viruses are first engulfed in the lumen of endocytic organelles and then become “activated” to access the intracellular cytoplasm. For enveloped viruses, the acidified, protease-enriched lumenal environments of the endosome cause conformational changes in viral surface proteins that induce virus-endosome membrane fusion and subsequent viral genome delivery. This general understanding of virus entry raised questions: do the HCoV-OC43 dependency factors identified by Poston et al. support only CoVs, or do they broadly support many different viruses taking endosomal cell entry routes? Poston et al. readily answered this question by challenging a collection of gene knockout (KO) cell lines with disparate viruses. There was strong CoV specificity: several dependency factors facilitated HCoVs but not several other unrelated endosome-entering viruses.

The discoveries that viruses differentially depend on specific endosome-associated proteins launched Poston et al. into more detailed mechanistic studies. With aims toward understanding how the endosomal proteins affect susceptibility to viruses, Poston et al. focused on vacuolar protein sorting 29 (VPS29), as its absence most potently suppressed several HCoVs while actually facilitating influenza virus infections. VPS29 is a multidomain regulatory subunit of both retromer and retriever complexes, and it also interacts with proteins controlling the RAB GTPases that coordinate vesicular formation and transport (5). To probe mechanisms by which VPS29 influences virus entry, Poston et al. used small interfering RNAs (siRNAs) to reduce levels of VPS29-interacting proteins and then evaluated sensitivities to HCoV and influenza virus infection. They also reconstituted VPS29 KO cells with mutant forms of VPS29 that cannot interact with retromer complexes. The gene knockdown and complementation experiments yielded concordant results implicating retromer complexes (VPS35/VPS29/VPS26) in facilitating coronavirus and restricting influenza virus infections.

This raised further questions about the distinct endocytic environments required for infection by HCoVs and influenza viruses and the specific ways that VPS29-containing retromer complexes provide favorable environments for CoV entry. Poston et al. approached these matters with high-quality subcellular imaging analyses. Cells lacking VPS29 had notably swollen intracellular endosomal vesicles. The vesicles readily incorporated extracellular cargo, but entrance of pH-sensitive fluorophores revealed that they were less acidic than normal endosomes. Extracellular cargo also included fluorescently tagged SARS-CoV-2 pseudovirus particles, which trafficked into vesicle interiors but, interestingly, remained durably inside, apparently unable to escape through fusion with the lipid bilayers delimiting the large vesicles. In sharp contrast, extracellular fluorescent influenza pseudovirus particles were similarly engulfed but did not accumulate within the enlarged endosomes of VPS29-deficient cells, consistent with rapid virus-vesicle membrane fusion and successful influenza virus entry.

These concordant observations established the VPS29 retromer in promoting CoV and restricting influenza virus infections, but they did not reveal the particular pro-CoV host factors that VPS29 might provide to endosomes. Yet proteases were considered the relevant host factors, as it is well-known that many CoVs require endosomal proteases, specifically cathepsin L, to proteolytically activate viral spike proteins for membrane fusion (6), and there were good reasons to suspect that VPS29 was needed to supply this protease activity. VPS29-containing retromer complexes may direct components needed to traffic lysosomal prohydrolases into endosomes. Therefore, Poston et al. performed additional imaging analyses and, perhaps surprisingly, found abundant cathepsin L proteins in endosomes irrespective of VPS29. This finding raised alternative possibilities. Having demonstrated that the swollen endosomes of VPS29-deficient cells were deacidified, Poston et al. questioned the cathepsin L enzyme activities within these vesicles, as acidification both generates and maintains lysosomal hydrolase enzyme activities. Imaging analyses clearly demonstrated very little cathepsin L enzyme activities. VPS29-associated complexes, therefore, likely support CoV entry quite indirectly by acidifying endosomes such that internalized viruses encounter enough active proteolytic enzymes to trigger their membrane fusion.

Of note, by complementing their molecular genetic methods with orthogonal pharmacologic approaches, Poston et al. showed that normal VPS29-containing cells exposed to cathepsin inhibitors closely reflected VPS29-deficient knockout cells in that they were resistant to SARS-CoV-2 and contained dramatically enlarged endosomes with SARS-CoV-2 particles entrapped therein. Together, the results indicated that elimination of VPS29 or direct cathepsin inhibition provides similar restriction of HCoV entry at the level of virus-endosome fusion activation.

Among the valuable features of the Poston et al. study is its movement beyond identification of host dependency factors and toward establishing their operating mechanisms. Their works highlight the retromer in facilitating or restricting distinct enveloped virus entry processes. Their findings indicate the potential to transiently disturb endosome vesicular trafficking or endolysosome maturation to selectively block virus entry. Yet there are several complicating features to this antiviral approach. Perturbed endosomal conditions suppressing one group of viruses can end up facilitating another group. Along these lines, it is not yet clear how the absence of VPS29 increases sensitivity to influenza viruses. Influenza viruses are acid activated for membrane fusion; there is no role for endosomal proteolysis. One possibility is that endosomes in VPS29-deficient cells are maintained at a “Goldilocks” pH for influenza, sufficiently acidic to trigger influenza virus membrane fusion (7) but not enough to activate potentially virus-degrading endolysosomal proteases. Several other hypotheses are credible, and it will be valuable to further distinguish the pro- and antiviral roles of endosomal protons and proteases in different infection contexts.

Additional complicating features come with the profound effects of both virus and cell context in determining requirements for endosomal factors. This is perhaps most evident in studies of the coronaviruses. Some CoV strains require aggressively proteolytic endosomal environments to infect new target cells. This is because their spike proteins do not readily display cleavage sites to fusion-activating proteases, often because they are not “primed” to expose these sites by prior severing at alternative locations. These viruses include the historical SARS-CoV that caused the 2002 to 2003 epidemic (6) and the cell culture-adapted HCoV OC43 that Poston et al. used to identify VPS29 and related endosomal dependency factors (8). Other CoV strains have spike proteins that are mostly “primed” and susceptible to fusion activation by proteases such as TMPRSS2, which are present on target cell surfaces. Consequently, these viruses do not require endosomal cathepsin-rich environments for infection (9). SARS-CoV-2 is evolving around these extremes, constantly adapting to different host cell environments in which spike-activating proteases vary in amount and subcellular position along the cell entry pathway. Indeed, Poston et al. showed that virus entry mediated by ancestral (2020) SARS-CoV-2 spikes was significantly less dependent on VPS29 than current (2022) Omicron variant spikes. There are relevant relationships between endosome dependence and virus tropism for target cells and organs (10), pathogenicity (11), and antiviral drug efficacies (12). It will be important to continue assessing SARS-CoV-2 for variations in endosome-specific host dependency factors as the virus becomes endemic in the human population.
